# Simple Field Assays to Check Quality of Current Artemisinin-Based Antimalarial Combination Formulations

**DOI:** 10.1371/journal.pone.0007270

**Published:** 2009-09-30

**Authors:** Jean-Robert Ioset, Harparkash Kaur

**Affiliations:** London School of Hygiene and Tropical Medicine, London, United Kingdom; East Carolina University, United States of America

## Abstract

**Introduction:**

Malaria continues to be one of the major public health problems in Africa, Asia and Latin America. Artemisinin derivatives (ARTs; artesunate, artemether, and dihydroartemisinin) derived from the herb, *Artemisia annua*, are the most effective antimalarial drugs available providing rapid cures. The World Health Organisation (WHO) has recommended that all antimalarials must be combined with an artemisinin component (artemisinin-based combination therapy; ACT) for use as first line treatment against malaria. This class of drugs is now first-line policy in most malaria-endemic countries. Reports of ad hoc surveys from South East Asia show that up to 50% of the artesunate currently sold is counterfeit. Drug quality is rarely assessed in resource poor countries in part due to lack of dedicated laboratory facilities which are expensive to build, equip and maintain. With a view to address this unmet need we developed two novel colour reaction assays that can be used in the field to check the quality of ARTs.

**Methods and Findings:**

Our assays utilise thin layer chromatography silica gel sheets and 2, 4 dinitrophenylhydrazine or 4-Benzoylamino-2, 5-dimethoxybenzenediazonium chloride hemi (zinc chloride) salt as the reagents showing a pink or blue product respectively only in the presence ARTs. We are able to detect as low as 10% of ARTs in ACTs (WINTHROP - artesunate/amodiaquine, Coartem^®^-artemether/lumefantrine and Duocortexcin - dihydroartemisinin/piperaquine). The assays have been validated extensively by testing eighty readily accessible and widely used drugs in malaria endemic countries. None of the other antimalarial drugs or a range of commonly used excipients, antiretroviral drugs or other frequently used drugs from the WHO essential drugs list such as analgesics or antibiotics are detected with our assays.

**Conclusions:**

Our two independent assays requiring no specialist training are specific, simple to use, rapid, robust, reproducible, inexpensive and, have successfully resulted in detecting two counterfeit drugs within a small scale screening survey of over 100 declared artemisinin-containing drugs collected from various Asian and African countries. These promising results indicate that the assays will provide a useful first test to assure the quality of the ACTs formulations in resource poor malaria endemic areas when there is an absence of dedicated medicines quality laboratory facilities.

## Introduction

Malaria continues to be one of the major public health problems in Africa, Asia and Latin America. *Plasmodium falciparum* (Welch, 1897) malaria is estimated to be the direct cause of 213.5 million clinical episodes in Africa and over 1 million deaths per year, mostly in children [1], [2]. New strains of the potentially fatal form of malaria, *Plasmodium falciparum,* have emerged that have developed resistance to most first- and second-line antimalarial drugs. Artemisinin derivatives (ARTs; artesunate  = AS, dihydroartemisinin  = DHA, artemether = AM, arteether = AE) derived from the herb, quing hao, sweet wormwood or *Artemisia annua* L (Asteraceae), are the most effective antimalarial drugs available providing rapid cures. The WHO has recommended that all antimalarials must be combined with an artemisinin component (artemisinin-based combination therapy; ACT) for use as first line treatment against malaria, and this class of drugs is now first-line policy in most malaria-endemic countries [3].

The increase in demand for ACTs, where the artemisinin derivative component is the expensive part, places these drugs at the inevitable risk of being counterfeited or produced at lower cost using substandard technology. Formulations may contain insufficient active ingredients, no active ingredients, the wrong (possibly toxic) ingredients, or fail to dissolve [4]. Additionally quality of the formulations may suffer as a result of being transported and stored inappropriately. Fake and substandard drugs are a great and growing problem.

There is evidence of the widespread distribution of fake artesunate tablets in SE Asia, resulting in fatalities among people who would have otherwise survived their malaria infection. Reports and studies suggested that there has been serious problem related to poor quality of antimalarial drugs in SE Asia, including countries in the Mekong sub-region emphasising Cambodia in particular [5], [6]–[10]. This problem has now spread to Africa as the market for ACTs expands [9]. Counterfeit artemisinin derivatives and combination therapies have already been described from 6 sub-Saharan African countries [11], [12], but to date no large scale efforts have been undertaken to assess their prevalence and this is principally due to lack of dedicated laboratory facilities.

Robust, inexpensive, portable, simple to conduct, rapid and reliable quality control methods to detect poor quality ARTs are not widely used as they are not readily available. The methods need to be reasonably accurate and can be carried out with the minimal use of toxic or flammable reagents. Dr Ying (Shanghai Institute of Materia Medica of the Chinese Academy of Sciences) asserts “If local drug regulators in developing countries are not equipped with easily portable, operable and low-cost analysis equipment, it is still very difficult for them to effectively and efficiently prevent fake drugs against malaria” [13].

To address an unmet need for a simple, reliable, affordable test that can be used at the point of screening with minimal training or chemical expertise we report here the results of two specific, simple to use, rapid, reproducible and cost-effective assays that we developed for the detection of ARTs not only in mono formulations but also in ACTs. These colorimetric assays utilise thin layer chromatography (TLC) silica gel sheets and 2, 4 dinitrophenylhydrazine or 4-Benzoylamino-2, 5-dimethoxybenzenediazonium chloride hemi (zinc chloride) salt as the reagents to detect the ARTs.

## Materials and Methods

### Reagents

Artemisinin and dihydroartemisinin were from Sigma-Aldrich (Dorset, UK), artesunate (kind gift from GSK) and artemether was from LGC Standards (Teddignton, UK) were used as reference compounds. Artemisone was a kind gift from Prof R Haynes, Laboratory of Chemistry, Hong Kong University. OZ 277 was a gift from MMV. The Dispro-1, 2, 4, 5-tetraoxane was a gift from Dr P O'Neil, Department of Chemistry, University of Liverpool, UK. Thin layer chromatography aluminium sheets Silica gel 60 F_254_ (Fluka product code: 60743) were from Sigma-Aldrich (Dorset, UK). Excipients were obtained from the Laboratoire de Galénique, School of Pharmacy, University of Geneva. Drugs from the WHO essential list were purchased from Sigma-Aldrich. Other antimalarials drugs are part of the compound library from the Pharmacology Lab at LSHTM. MininLab^®^ was purchased from GPHF, Germany.

The reagent 2, 4 dinitrophenylhydrazine (Brady's reagent, DNP) was purchased as a powder from Tokyo Chemical Industry UK Ltd, Oxford, OX4 4GA United Kingdom and the solution (∼0.005 M in ethanol) was from Fluka (product code: 18189).

The reagent 4-Benzoylamino-2, 5-dimethoxybenzenediazonium chloride hemi (zinc chloride) salt (Fast Blue RR salt, FBS) was obtained from Sigma-Aldrich (Fluka product code: 44680).

DNP is sold as a wet reagent as it is harmful when dry and toxicological hazards of the FBS have not been fully investigated. Our assays are conducted with the drop wise addition of a dilute solution of these reagents thus minimising the hazards.

### Methods and Results

The principal of the assays involves dissolving the reference standard or a pulverized (with a pestle) tablet in methanol, mixing well and applying a known amount as a spot in a graphite pencil drawn circle on the TLC sheet followed by dropping either of the two reagents onto the spot and allowing the reaction to proceed at room temperature. We tested various supports (such as alumina coated on plastic sheets, filter paper [Whatman^®^ product code 1001 125, qualitative, Grade No. 1 - Medium flow rate, medium porosity, with 11 µm particle retention size]). The most definitive results in terms of limit of detection by depth of colour seen and reproducibility were achieved using TLC aluminium sheets Silica gel 60 F_254_.

A practical extension of this work is that the identity of each ART can be determined due to varying polarity. This is achieved by allowing the spotted sample to be drawn up the TLC sheet via capillary action using a mixture of chloroform: methanol: acetic acid (98∶2∶0.1) described later in this article.

#### Preparation of samples

Tablets of co-formulations (amodiaquine/artesunate or dihydroartemisinin/piperaquine or artemether/lumefantrine) were pulverised with a pestle, methanol (2 mL) added and solubilisation achieved by placing the mixture in an ultrasonic bath for 30 s. The mixture was then left on the bench for a couple of minutes to encourage sedimentation. The reference compounds (AS, AM, DHA, AE and artemisone: 2.5 mg) were dissolved in methanol (200 µL). The solutions were mixed thoroughly (manual shaking or vortex or ultrasonic bath) for 30 s.

#### Preparations of reagents to spray the plates

DNP reagent: 2,4-dinitrophenylhydrazine was completely dissolved in methanolic (80%: w/v) hydrochloric acid (1 M) solution yielding a final concentration of 0.5 mg/mL.

FBS reagent: Fast Blue RR salt was prepared in water at 5.0 mg/mL and subsequently mixed 1∶1 with 80% methanolic 2M sulphuric acid (H_2_SO_4_) solution yielding a final concentration of 2.5 mg/mL.

#### Targets set for the tests and results

The assays that we developed selectively detect artemisinin derivatives in formulations. Our assays use commercially available compounds and TLC sheets or filter paper to produce two distinct chromophores depending on the reagent used, i.e. a pink colour results with DNP or a blue colour with FBS. These assays were adapted in consideration of the modest resources available in the developing world. Considerations taken into account prior to developing these assays are shown in Table 1 (constructed by JR Ioset and H Kaur). This table includes both target and minimum acceptable limits. The assays were designed to be used where there is an absence of specialised laboratories or trained staff.

**Table 1:** Product profile and minimum acceptable profile assay results.

Criteria	Target	Minimum acceptable	DNP test	Fast Blue test
Specificity	100%	98% and no interference with any antimalarials	98–100% no interference with antimalarials	99% no interference with antimalarials
Limit of detection*	<1 µg/mL	<100 µg/mL	1–10 µg (TLC) 50–100 µg (FP)	1–10 µg (TLC) 50–100 µg (FP)
Robustness	Not altered by changes in temp of 10°C	Not altered by small changes in temp 5°C	Consistent	Consistent
Quantitative aspect	Quantitative (±5%)	Qualitative (±20%)	Semi-quantitative (TLC)**	Semi-quantitative (TLC)**
Time needed for assay	Less than 5 min	Less than 15 min	5–10 min	5–10 min
Time needed for result	Instantaneous	Less than 8 hours	30–60 min	2–3 h
Amount of material needed for testing	Less than 1 mg	10 mg	2.5–5 mg	2.5–5 mg
Test support	As many as possible	At least a very cheap one	TLC and filter paper	TLC and filter paper
Cost of single assay	Less than 2 pence	10 pence	consistent	consistent

***Note:*** **TLC  =  Thin Layer Chromatography sheet; FP  =  Filter paper.**

* **Least amount of substance needed to be detected by the assay, LOD.**

** **Both qualitative and quantitative results will be needed:**

**– By definition – counterfeit ARTS preparations don't contain the active ARTS ingredient and it is most unlikely that one artemisinin derivative will be substituted by another.**

**- By definition – substandard drugs may contain a lower dose of active ingredient, may contain ARTS degradation products or be subjected to other lacks of conformity due to poor good manufacturing practice.**

### Validation

A complete validation of the two developed assays was undertaken in our laboratory following a systematic evaluation:

1. Tested for specificity and validated against the other available field method (Fast Red Test from GPHF-MiniLab^®^) [14]. This includes testing of all major antimalarials currently used on the market, a wide selection of active principles from the WHO list of essential drugs as well as a wide range of excipients used in the pharmaceutical formulation of tablets.
2. Application of the tests to a representative range of tablets from the field.
3. Confirming the results against high performance liquid chromatography with photo-diode array detection (HPLC-PDA) as the gold standard method.

We compared the results of our assays in parallel with the other available field test (Fast Red Test in the MiniLab^®^) [14] and found that our assays specifically detect the artemisinin component only. Our assay is therefore robust, reproducible and sensitive. It has also been extensively validated using HPLC-PDA the “reference standard method” to compare our assay. Our assays provide two user friendly/field friendly methods to monitor the quality of ARTs and ACT formulations for use in the developing countries.

#### Determining the specificity of the tests

We compared the results of our assays with the published colorimetric assay [15], [16] and established that our assays specifically detect the artemisinin derivatives clinically used as antimalarial drugs. None of the other antimalarial drugs or a range of commonly used excipients, antiretroviral drugs or other frequently used drugs from the WHO essential drugs list such as analgesics or antibiotics are detected with our assays. It is of special interest to note that artemisinin as such is not detected in either of our assays.

#### Other antimalarial drugs

Eleven of the thirty non-ARTs antimalarial formulations produced a pale to intense yellow, orange or red colour when tested using the Fast Red Test from the GPHF-MiniLab^®^ [14]. Additionally the antimalarial compounds - amodiaquine, mepacrine, primaquine, doxycycline, pyronaridine, atovaquone and lumefantrine produced the same shade of yellow colour that results with ARTs. The validation of the DNP and FBS assays was conducted in parallel with the published methods devised by Green (both unmodified version for the specific detection of artesunate [15] and modified version for the detection of artesunate, artemether and DHA [16]).

These drugs did not produce a colour when tested with our assays with the exception of primaquine which produced an orange-brown coloration only when FBS was used as a reagent and filter paper as the support. This potential confusion is overcome when TLC and the reagent FBS are used instead of filter paper as an unambiguous orange colour is produced instead of the expected blue.

#### Excipients

**Figure 1:**
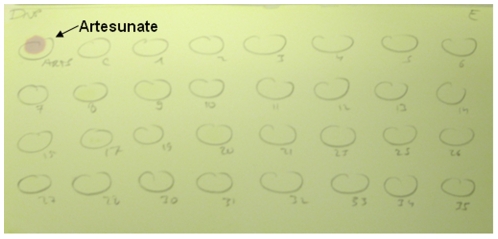
Shows that only artesunate gives a pink colour when DNP was sprayed onto the spotted artesunate and excipients onto TLC plate.

A total of thirty excipients were tested for their potential to react with DNP or FBS to result in the pink or blue colours that indicate the presence of the ARTs. Most of these excipients were selected following expert advice (Dr Pascal Furrer, University of Geneva) from the Handbook of Pharmaceutical Excipients for their likelihood to be used in the formulation of tablets, especially the ones manufactured by direct compression of the material [17]. Furthermore the selected powders are readily available, cheap and can be easily used to manufacture counterfeit tablets. A total of seven excipients produced a yellow or orange reaction using the Fast Red Test from the GPHF-MiniLab^®^ [14]. None of the excipients was found to react with either of the reagents used in our assays and Figure 1 is typical of the results achieved (quality of the photograph shown is poor).

#### Drugs other than ARTs from WHO essential drugs list

Eighty readily accessible and widely used drugs in malaria endemic countries were selected from the WHO list of essential drugs. This selection process ensured that most of the drugs chosen included the ones that can accidentally or intentionally be sold as ARTs; other criteria used included testing drugs that are affordable and have therapeutic efficacy to treat other infectious diseases in endemic countries. The mode of administration was also considered as oral drugs were preferentially selected. Of the 80 drugs selected, 29 were not only tested as pure compounds but also as pharmaceutical formulations (capsules or tablets) supplied as part of the GPHF-Minilab^®^.

A total of 24 compounds (19 different formulations) were tested following the experimental conditions described in the GPHF-Minilab^®^ using the Fast Red test [14], [16] and yielded not only yellow to orange colours but also red or brown or pink that can hinder the detection of ARTS. Additionally a total of 22 samples (17 drugs) produced ambiguous coloured reactions (yellow, green or orange colours). A number of samples are yellow to orange in colour (17 compounds; 15 formulations) before addition of the reagents hence not suitable for testing using the Fast Red test in the GPHF-Minilab^®^ as they give false positive results.

Application of our assays to these 80 drugs showed that 4 drugs produced a colour only with FBS reagent: brown colourations with furosemide and amphotericin B, violet coloration for silver nitrate and blue colouration for erythromycin. Hence, erythromycin is the only compound that reacts in a similar way to ARTS yielding a blue coloration on both TLC plate and filter paper. The DNP test does not produce a colour with erythromycin hence ensuring the specificity of one of the two assays that we developed. All other active principles, excipients and formulated drugs tested in the DNP assay, only a pale orange to red coloration was observed with dexamethasone (reacts immediately), chlorpromazine, and hydrocortisone. Whereas a grey-violet spot was observed with silver nitrate TLC but could unambiguously be discriminated from the specific colour reaction obtained with ARTS products.

#### Chromatographic separation of ARTs on TLC and on HPLC (qualitative vs. quantitative analyses)

A useful extension of this work is that the identity of the ARTs can be determined based on their varying polarity. This is achieved by allowing the spotted sample to be drawn up the TLC plate via capillary action. The solution under investigation is placed as a spot on the TLC sheet and allowed to elute when placed in the solvent which travels up the sheet as a result of capillary action. The ARTs travel at different rates due to differences in their solubility in the solvent, and due to differences in their attraction to the silica gel. Following this elution step the colourless ARTS are detected by spraying with either a methanolic solution of DNP or FBS which produce the coloured spot.

#### Modus operandi for the chromatography on thin layer sheet

**Figure 2:**
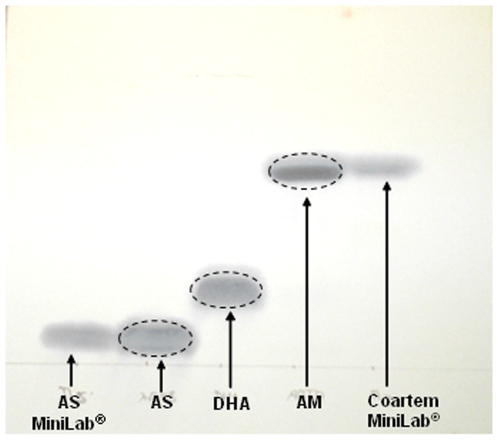
TLC chromatogram of the ARTs; showing the different rates of travel by artesunate (AS) reference standard and sample tablet from MiniLab^®^, dihydroartemisinin (DHA) and artemether (AM) reference standard and sample tablet from MiniLab^®^ (Coartem) on silica gel sheets using chloroform: methanol: acetic acid 98:2:0.1 as an elution system.

**Figure 3:**
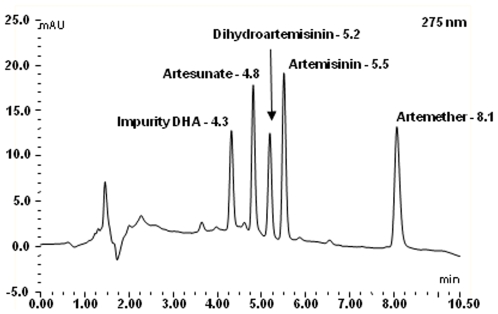
Shows the HPLC separation of reference compounds; artemisinin (5.5 min), artesunate (4.8 min), dihydroartemisin (5.2 min) and artemether (8.1 min).

A small spot of solution containing the compound was applied to the TLC sheet, about one centimetre from the base. The sheet was placed in the tank such that the solvent mixture (chloroform: methanol: acetic acid [v/v: 98∶2∶0.1]) was below the level of the spotted solution and then travels up the sheet. The sheet was taken out of the solvent before it reaches the top of the sheet, allowed to dry at room temperature and, sprayed with either the DNP or FBS reagent to detect the ARTs that migrated at different rates on the sheet as shown on Figure 2. The HPLC separation of these ARTs is shown in Figure 3 demonstrating that the techniques are complementary.

#### High Performance Liquid Chromatographic (HPLC) analyses

Separation of the ARTs and artemisinin (Figure 3) was achieved using HPLC equipment and software from Dionex (UK) Ltd, Camberley, Surrey, U.K. Separations were carried out utilising a GENESIS AQ 4 µm column (150×4.6 mm, GRACEVYDAC^®^, Alltech Associates Applied Science Ltd, Lancashire, UK) eluting with ammonium formate (20 mM, pH 2.7) and acetonitrile (v/v; 60∶40 to 85∶15 over 7.0 min) passing through the UV-photo-diode array detector (UV-PDA; Dionex model PDA-100) set at 275 nm at a flow rate of 1.0 mL/min. Peak identity was confirmed by measuring the retention time, spiking the sample with additional amounts of each compound and determination of absorbance spectra using the UV-PDA. A calibration curve of ARTs was generated by Chromeleon (Dionex software) using known amounts of the standard (0–10 mg/mL) in methanol. Similarly a calibration curve of amodiaquine (0–200 mg/mL; but diluted 1∶10 to inject on column) was generated.

### Applications of our assays to test the quality of retailed formulations

#### Modus operandi for the spot test to check for the artemisinin derivative in a coformulation of amodiaquine/artesunate

**Figure 4:**
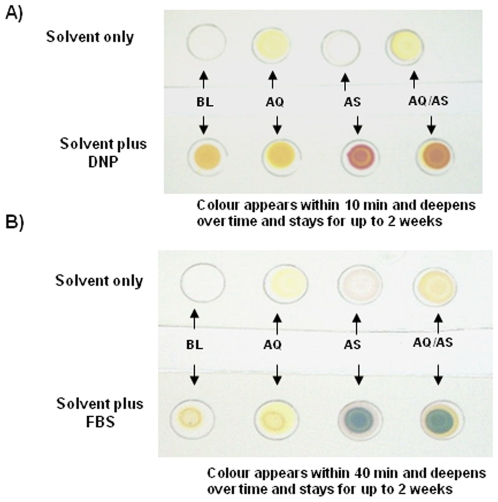
Shows TLC results of analysing the ACT- AS/AQ; with A) DNP and B) FBS as the reagents. Each result was compared with using solvent alone (1 M HCl for A the DNP test and 2 M H_2_SO_4_ for B the FBS test). BL  =  methanol, AQ  =  modiaquine, AS  =  artesunate and AS/AQ  =  the ACT artesunate plus amodiaquine such that each AS spot is 5 mg and each AQ spot is 15 mg.

**Figure 5:**
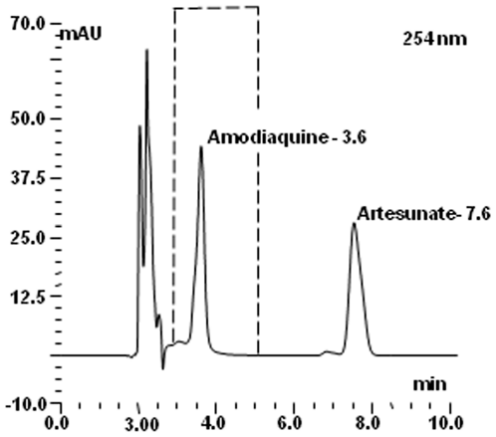
Shows the HPLC separation of reference standards of amodiaquine (3.6 min) and artesunate (7.6 min). The dotted line indicated the peak for amodiaquine when a formulation of AS/AQ is tested.

A tablet of the co-formulation amodiaquine/artesunate (150 mg AQ/50 mg AS) was crushed and dissolved in methanol (10 mL). Circles were drawn on the TLC sheet using a graphite pencil. The solution of the reference compound and of the co-formulation in methanol (10 µL) was then applied onto the circle on using a micropipette or a graduated capillary. The solution was applied slowly to concentrate the sample in the given area and each spot is 5 µg AS and 15 µg AQ, respectively or in the co-formulation. Two reagents were evaluated as follows:

DNP solution (5 µL; ∼0.005 M in ethanol) or FBS solution (10 µL; 5 mg in 2 M H_2_SO_4_) were each dropped onto individual spots and allowed to stand at room temperature. Solvents (acid solution without the reagents) were also dropped onto the spots and methanol was used as the blank. Figure 4 shows the results as DNP gave a pink colour on the TLC after 10 min, whereas FBS gave a blue colour after 40 min with intensity varying in function of the concentration of the ART detected. The depth of colour was stable for up to 2 weeks. The methanolic solution of AQ/AS was also analysed using the HPLC method described above to check the amounts of each component in this co-formulation (Figure 5).

#### Determining the detection limit of the assays for testing the amount of ARTs in co-formulations

**Figure 6:**
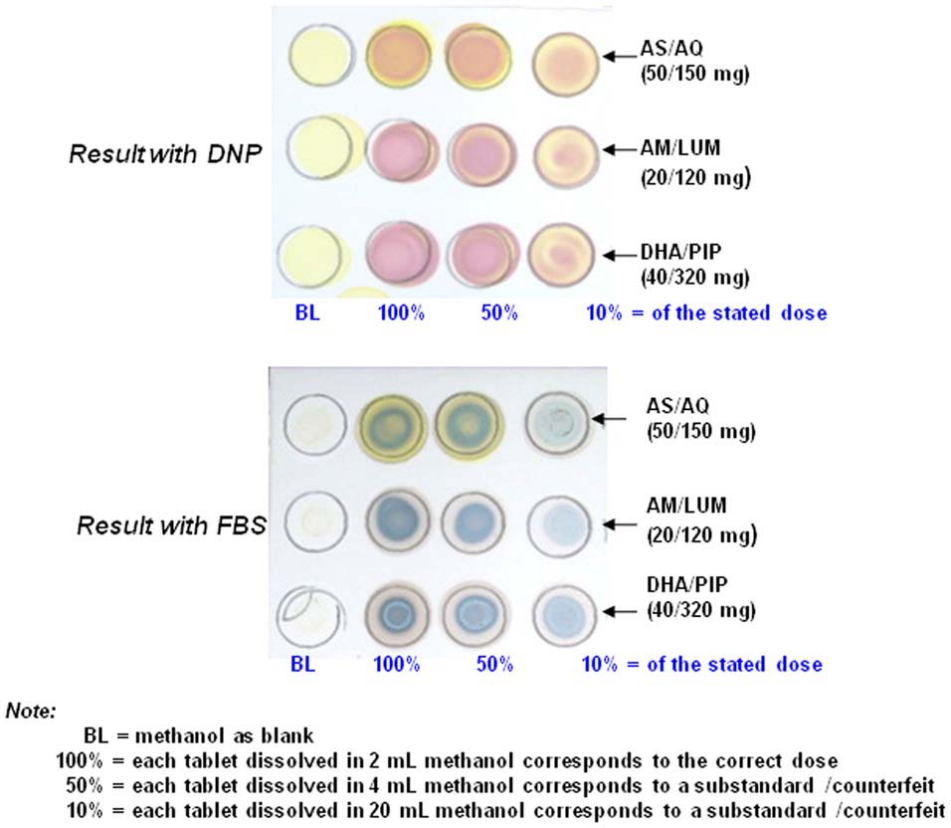
Shows the detection of the artemisinin component from 100% (which is the tablet containing the correct amount of the active principal ingredient as stated on the packet) down to 50% and 10% (which will imply that the product is substandard or counterfeit) in formulations of artesunate/amodiaquine (AS/AQ;100%  =  50 mg, 50%  = 25 mg and 10%  =  5 mg), artemether/lumefantrine (AM/LUM, Coartem^®^; 100%  =  20 mg, 50%  = 10 mg and 10%  =  2 mg), and dihydroartemisinin/piperaquine (DHA/PIP, Duocortexin^®^; 100%  =  40 mgs, 50%  = 20 mg and 10%  =  4 mg).

The limits of detection for the co-formulations (WINTHROP - artesunate/amodiaquine, Coartem^®^-artemether/lumefantrine and Duocortexcin - dihydroartemisinin/piperaquine) was determined by pulverising each representative tablet and dissolving in methanol (2 mL). Further dilutions were carried out to achieve 50% and 10% solutions and 10 µL of each solution was then applied to the TLC plate followed by either DNP or FBS solutions (10 µL each) and the resulting colour is shown Figure 6. The depth of colour with DNP was achieved within 10 min and with FBS was achieved within 40 min, and for both the depth of colour was stable for up to 2 weeks.

### Detection of suspect formulations collected from various countries

**Figure 7:**
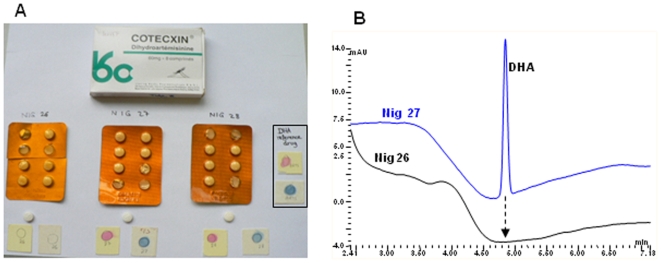
Shows the analyses of Cotexcin^®^ samples collected from Nigeria. A) Showing the detection of a fake sample of Cotexcin^®^ using our assays (sample Nig 26 did not give the pink or blue colour with DNP or FBS on TLC when compared with samples Nig 27 and 28 as well as DHA, the reference active principal ingredient - on the far right hand side). B) Shows the HPLC chromatogram showing the presence of the DHA peak in samples Nig 27 and 28 but its absence in sample Nig 26 indicated by the dotted arrow at around 4.9 min.

Samples of more than 150 artemisinin-based formulations were collected by our colleagues (working on varying aspects of malaria research) from 13 countries in Asia, South America and Africa to check for quality using our assays. Of these samples two were found not to contain the stated active ingredient: hence they are suspect (Figure 7A, shows the detection of suspect DHA tablets on sale in Nigeria). Our attempts to obtain further samples of these tablets from Nigeria were not successful. We therefore purchased 21 packets (8 tablets in each) of DHA under the brand names Cotecxin^®^ and Alaxin^®^ (manufactured in China and India respectively) from varying outlets in Nairobi, Kenya. Though these packets varied in price (210 to 390 Kenyan Shillings) the tablets were found to contain the stated amounts of the active ingredient.

The other sample which did not give the pink or blue spots in our assays was from Vietnam which did not contain artesunate the stated active ingredient.

The quantitative analysis of each tablet was subsequently carried out using our HPLC method (see the chromatogram; Figure 7B, above) which indicated that the tablets that failed to produce colour in our assays did not contain the active component, DHA.

## Discussion

The ability to identify counterfeit or poor quality formulations is the crucial component of a drug quality assurance system. Many disease endemic countries do not have regulatory mechanisms or the technology at their disposal to ensure that the drugs in circulation are of good quality thus leaving their populations vulnerable to the risk of counterfeit and substandard drugs. Two factors prevent the detection of fake anti-malarials at an early stage of market penetration when intervention has the greatest chance of success. The first is not having the right tools to be able to detect counterfeit drugs at the point of screening. The second is the lack of routine systematic nationwide drug sampling from a complete range of drug retailers in resource poor countries.

Several analytical techniques using high tech equipment including liquid chromatography-mass spectroscopy, and high performance liquid chromatography coupled with photo diode array detection or electrochemical detection have been developed to detect and quantify artemisinins in formulations or biological fluids. These methods have several merits such as accuracy, repeatability, specificity and precision but they are expensive, require access to sophisticated equipment and expertise and therefore cannot be implemented for routine analysis in the field.

Detection of ARTs on thin layer chromatography (TLC) by different reagents including iodine, concentrated sulphuric acid or 4-methoxybenzaldehyde [18] has been reported. However these methods are not specific for ARTS as other compounds react under the stated experimental conditions resulting in the same or similar colours. So far one field assay to detect and authenticate ARTs has been developed by Green *et al.* (Centre Diseases Control, Atlanta, USA) and marketed by German Pharma Health Fund (GPHF-MinliLab^®^) [14]–[16]. It is a test tube method that relies, on the reaction of a commercially available diazonium salt, Fast Red TR salt, with the ARTs leading to the formation of a yellow coloured solution which limits its use, especially for ACTs when the co-formulated antimalarial is itself yellow (amodiaquine, atovaquone, primaquine, lumefantrine, pyronaridine and doxycycline) and hence give a false positive result.

We have described two colour reaction assays that are specific, easy to use, robust, inexpensive and rapid, that only detect the artemisinin component of the formulation and can be used to spot check the quality of ACTs in the field. Our assays use commercially available reagents DNP and FBS dissolved in strong acids. Reaction of the resultant solutions with the ARTs result in distinctly coloured spots (pink with DNP and blue with FBS). The experimental conditions required to achieve these results and the lack of an immediate coloured reaction after pulverisation of the tablet in DNP or FBS solutions suggest that a decomposition product of ARTs is involved in the production of the coloured product. The acid or base transformation of ARTs has been reported to result in enolate and carboxylates or α, β- unsaturated decalones [19], [20]. It was demonstrated experimentally that diluted solutions of these mineral acids did not produce the colour nor do stronger acids including nitric acid and phosphoric acid.

Importantly, amounts lower than 10 mg/mL of artemisinin itself do not yield a coloured product on TLC in either of the assays. Artemisone, another semi-synthetic artemisinin clinical candidate, did react positively in both tests. However, the tetraoxanes (cyclic peroxides) OZ 277 and dispro-1, 2, 4, 5-tetraoxane did not produce the anticipated pink and blue colours.

It is known that DNP reacts with carbonyl compounds in the presence of an acid catalyst to produce a coloured product referred to as a 2,4-dinitrophenylhydrazone. ARTs do not possess a free carbonyl group (except in the case of the parent artemisinin which has a lactone carbonyl that does not react with DNP). ARTs are structurally complex compounds and may undergo a series of reactions under our acidic experimental conditions to expose hydroperoxide and carbonyl groups both of which may lead to the pink and blue coloured products with the reagents DNP and FBS respectively. However we have found that simple hydroperoxides such as *tert*-butyl hydroperoxide do not produce the pink or blue chromophore. Our attempts to characterise the chemical reaction mechanisms for the results have indicated a need for in depth chemical investigation which is beyond the scope of our present resources.

The validation of the DNP and FBS assays was conducted in parallel with the published methods devised by Green (both unmodified version for the specific detection of artesunate [15] and modified version for the detection of artesunate, artemether and DHA [16] and sulphuric acid assay [10]).

Limits of detection of the ARTs compounds with the reagents DNP and FBS in the assays where carried out on TLC sheet as well as filter paper. A greater amount (2–3 times) of ARTs was necessary to detect an unambiguous depth of colour on filter paper compared to that achieved on the TLC sheet. This is probably due to a larger diffusion and absorption of the compound by the filter paper. We are able to detect as little as 10% of the declared active principal ingredient, as stated on the packet, in formulations of AS/AQ, AM/LUM and DHA/PIP which suggests the DNP and the FBS. These assays can not only be used to identify counterfeit medicines but also to get a semi-quantitative measure of the amounts of ARTS in formulations seen by the depth of colour (see Figure 6). When less than the stated dose is detected the formulations should be deemed suspect and not used until analysed using a medicines quality laboratory which is equipped with the necessary chromatography systems.

The identity of ARTS can be unambiguously determined by running a TLC migration with the elution system described above (see Figure 3). Both DNP and FBS reagents can be used for the detection of ARTs. Issue of formulations quality can be further complicated by the fact that exposure of good quality drugs to light, heat, and humidity through inadequate storage may result in deterioration and complicate their classification as counterfeit or substandard. Degradation of rectal artesunate capsules have been reported to produce DHA, peroxyhemiacetal and deoxyartemisinin as a result of aging [21]. We can detect DHA in our assays and the possibility of DNP and FBS giving the pink and blue chromophores with the other degradation products will be investigated when we acquire them. If the decomposition products of ARTs [21], were to react with both chemicals, and then separating the components with migration on TLC will serve to indicate the quality of the formulations.

Field assays need to be robust as they will inevitably be subject to changes during their use. Both DNP and FBS are stable reagents that may be stored at room temperature for longer than 6 months. Solutions of DNP and FBS gave the expected results when kept for 1–2 months at 20°C in our lab. Samples preparation is a straight forward process that does not leave much room for experimental variations. Assays should be conducted using a reference ARTs drug (not artemisinin) and a negative control (methanol used for extraction of ARTs in samples) as shown in Figures 4 and 6.

The assays have been used successfully to identify two samples which did not contain the stated artemisinin derivative component (one shown in Figure 7A) from a randomly selected survey of ARTs tablets collected in various malaria endemic countries - South East Asia and Africa (Vietnam, Nigeria, Tanzania, Kenya, Burma, Venezuela and China). These results were corroborated with HPLC analyses used to confirm the presence or absence of artemisinin derivative component in the formulations (Figure 7B).

### Conclusion

ACTs are recommended by WHO as first line treatment for malaria and have been widely implemented in numerous countries to date. The generalised use of ACTs in conjunction with their outstanding therapeutic value and high price has put them at risk of being counterfeited or produced at facilities that are not accredited. We recently reported on the poor quality of non-ACT antimalarials from a nationwide study in rural Tanzania [22]. Antimalarial drugs purchased through the retail sector are one of the key tools used by poor African households to control malaria. ACTs are expensive and create a significant economic burden on families so it is essential that the quality and safety of these medicines is assured. However, drug quality is rarely assessed in resource poor countries in part due to lack of dedicated laboratory facilities which are expensive to build, equip and maintain. The absence of simple, quick, cost effective and reliable tests to detect fake and substandard products can only facilitate the spread of suspect ACTs.

We have demonstrated two new robust assays [23] that have successfully resulted in detecting two counterfeit drugs within a small scale screening survey of over 100 declared artemisinin-containing drugs collected from various Asian and African countries.

These promising results indicate that in a kit format the assays we have developed provide a useful first test to assure the quality of the ACTs in resource poor malaria endemic areas and suspect formulations can then be sent to medicines control laboratories for comprehensive investigation using state of the art equipment.
